# S09 Insights into the development of the physical activity environment policy index, a tool for benchmarking implementation of public policies to create healthy physical activity policy environments

**DOI:** 10.1093/eurpub/ckac093.044

**Published:** 2022-08-29

**Authors:** Catherine Woods, Joanna Zukowska, Sarah Forberger, Enrique Garcia, Peter Gelius, Anna Gobis, Liam Kelly, Piotr Krajewski, Jeroen Lakerveld, Sven Messing, Nicole denBraver, Kevin Volf

**Affiliations:** 1 Physical Activity for Health Research Cluster, Physical Education and Sport Sciences, University of Limerick, Limerick, Ireland; 2 Faculty of Civil and Environmenatl Engineering, Gdansk University of Technology, Poland, Gdansk, Poland; 3 Leibniz Institute for Prevention Research and Epidemiology, Leibniz Institute for Prevention Research and Epidemiology, BIPS, Germany, Bremen, Germany; 4 Sport Ireland, Sport Ireland, The Courtyard, Blanchardstown, Dublin, Dublin, Ireland; 5 Friedrich-Alexander-Universitat, Friedrich-Alexander-Universität Erlangen-Nürnberg, Erlangen, Germany, Erlangen, Germany; 6 VU University Amsterdam, Department of Epidemiology and Data Science, Amsterdam Public Health research institute, De Boelelaan 1089a, Amsterdam, the Netherlands., Amsterdam, The Netherlands

**Keywords:** physical activity, benchmarking, policy, implementation, sport, transport, mass media

## Abstract

The UN Sustainable Development Goals' (https://sustainabledevelopment.un.org/) and the Global Action Plan on Physical Activity (https://www.who.int/ncds/prevention/physical-activity/gappa) highlight the need to move beyond individual behaviour change to broader policy and system approaches, focusing not only on health but also on sustainability. Recently, policy responses to the epidemics of physical inactivity and sedentary behaviour have grown. The Global Observatory for Physical Activity (GoPA) reports that by 2013, 139 countries were members of its physical activity advocacy alliance and 26.6% of these countries had already published a stand-alone physical activity plan. The ‘Policy Evaluation Network' is a multi-disciplinary European research network aimed at understanding the impact of public policy for promoting healthy lifestyles in an effort to prevent non-communicable disease. To understand the progress governments are making in creating healthy policy environments, the benchmarking of best practice has proven effective for advancing the food policy agenda; however its usefulness for physical activity requires evaluation. Individual country results promote mutual learning between countries. This symposium will take a deep look at public policy in physical activity. It will summarise evidence from systematic literature reviews and present a tool for benchmarking progress. It will also discuss the potential next steps for addressing the inactivity, obesity and climate challenges through policy solutions in a systematic way.

The Chair will give an overview of context within which this symposium takes place e.g. GAPPA, Policy developments etc. They will also explain the symposium's purpose and objectives, introduce the speakers and direct questions.

Symposium Objectives

1. To review evidence for the contribution of transport, sport and mass media policy to the promotion of physical activity.

2. To show how evidence generate in each of these reviews contributed to the development of the physical activity environment policy index (PA-EPI).

3. To discuss the next steps for addressing inactivity by using policy intervention as a tool to catalyse change.

Abstract 1: Which transport policies increase physical activity of the whole of society? A Systematic Review.

Abstract 2: The impact of mass-media campaigns on physical activity: a review of reviews through a policy lens.

Abstract 3: Evidence of the impact of Sport Policies on physical activity and sport participation: A Systematic Mixed Studies Review.

Abstract 4: The development of the Physical Activity Environment Policy Index (PA-EPI): a tool for monitoring and benchmarking government policies and actions to improve physical activity.

The Discussant summarizes the presentations and provides insights on the specific topic area, generating an interactive discussion with the audience for at least 15 minutes, moderated by the Chair. (15 minutes) Conclusions: Some discussion of the possible contribution to, or implications for, the advancement of HEPA related goals.

